# *Flourensia cernua*: Hexane Extracts a Very Active Mycobactericidal Fraction from an Inactive Leaf Decoction against Pansensitive and Panresistant *Mycobacterium tuberculosis*

**DOI:** 10.1155/2011/782503

**Published:** 2011-03-31

**Authors:** Gloria María Molina-Salinas, Luis Manuel Peña-Rodríguez, Benito David Mata-Cárdenas, Fabiola Escalante-Erosa, Silvia González-Hernández, Víctor Manuel Torres de la Cruz, Herminia Guadalupe Martínez-Rodríguez, Salvador Said-Fernández

**Affiliations:** ^1^División de Biología Celular y Molecular, Centro de Investigación Biomédica del Noreste, IMSS, 64720 Monterrey, NL, Mexico; ^2^Grupo de Química Orgánica, Unidad de Biotecnología, Centro de Investigación Científica de Yucatán, 97200 Mérida, YU, Mexico; ^3^Facultad de Ciencias Químicas, Universidad Autónoma de Nuevo León, Monterrey, NL, Mexico; ^4^Departamento de Bioquímica y Medicina Molecular, Facultad de Medicina, Universidad Autónoma de Nuevo León, Monterrey, NL, Mexico

## Abstract

The efficacy of decoction in extracting mycobactericidal compounds from *Flourensia cernua (Hojasé)* leaves and fractionation with solvents having ascending polarity was compared with that of (i) ethanol extraction by still maceration, extraction with a Soxhlet device, shake-assisted maceration, or ultrasound-assisted maceration, followed by fractionation with *n*-hexane, ethyl acetate, and *n*-butanol; (ii) sequential extraction with *n*-hexane, ethyl acetate, and *n*-butanol, by still maceration, using a Soxhlet device, shake-assisted maceration, or ultrasound-assisted maceration. The *in vitro* mycobactericidal activity of each preparation was measured against drug-sensitive (SMtb) and drug-resistant (RMtb) *Mycobacterium tuberculosis* strains. The results of which were expressed as absolute mycobactericidal activity (AMA). These data were normalized to the ΣAMA of the decoction fraction set. Although decoction was inactive, the anti-RMtb normalized ΣAMA (NAMA) of its fractions was comparable with the anti-RMtb NAMA of the still maceration extracts and significantly higher than the anti-SMtb and anti-RMtb NAMAs of every other ethanol extract and serial extract and fraction. Hexane extracted, from decoction, material having 55.17% and 92.62% of antituberculosis activity against SMtb and RMtb, respectively. Although the mycobactericidal activity of decoction is undetectable; its efficacy in extracting *F. cernua* active metabolites against *M. tuberculosis* is substantially greater than almost all pharmacognostic methods.

## 1. Introduction

In bioassay-directed fractionation of plant extracts to isolate molecules that have activity against *Mycobacterium tuberculosis* or other bacteria, it is common to obtain inactive decoctions and infusions [1–4]. Conversely, aqueous preparations of plant extracts are the most active, harboring antioxidant [5], anti-inflammatory [6], antidiabetic [7], cytotoxic [8], and immunomodulatory [9] properties. 

Due to this disparity, aqueous preparations are typically skipped when new antituberculosis or antibiotic principles are purified from a plant. Bioassay fractionation is generally started with a polar organic solvent, such as ethanol or methanol [10]. In contrast, through the ages, the efficacy of plant decoctions in treating many respiratory and infectious ailments has been highly touted. Thus, omitting the aqueous extraction step in antituberculosis bioassay fractionation of a particular plant should be examined more carefully, because such an action might produce false-negative results, wherein new, potentially valuable antituberculosis molecules are discarded.

Decoction of *Flourensia cernua* (*Hojasé*) is a component of Mexican traditional medicine. In northern Mexico, its leaves are used as a decoction to treat indigestion, as an expectorant and as a cure for respiratory infections, including tuberculosis [11].

We investigated (i) whether decoction generates active fractions against *M. tuberculosis* using solvents that have disparate polarities; (ii) whether any of the four most recognized pharmacognostic methods (still maceration, extraction with a Soxhlet device, shake-assisted maceration, and ultrasound-assisted maceration) and fractionation with *n*-hexane, ethyl acetate, and *n*-butanol; or sequential extraction with *n*-hexane, ethyl acetate, and *n*-butanol, using still maceration, a Soxhlet device, shake-assisted maceration, and ultrasound-assisted maceration is more efficacious than decoction in yielding preparations from *F. cernua* leaves that are rich in antituberculosis activity; (iii) whether such extracts are active against *M. tuberculosis* strains that are sensitive or resistant to the five first-line antituberculosis medications.

## 2. Methods

### 2.1. General Procedures

Organic solvents, reactive-grade *n*-hexane (*n*-Hex), ethanol (EtOH), ethyl acetate (EtOAc), *n*-butanol (*n*-BuOH), and methanol (MeOH) were purchased from Productos Químicos Monterrey (Monterrey, NL México). Dimethyl sulphoxide (DMSO) and the antibiotics rifampin and ofloxacin were supplied by Sigma-Aldrich Chemical Co., St. Louis, MO, USA.

### 2.2. Plant Material

Whole *Flourensia cernua* plants (2 kg) were collected by Juan Antonio Luna de la Rosa at Galeana, Nuevo León, México, in October, 2006 and authenticated by the biologist María Consuelo González de la Rosa; a voucher specimen (voucher no. 024027) has been deposited at the herbarium of Facultad de Ciencias Biológicas, Universidad Autónoma de Nuevo León. The plant material was dried in an oven (J.M. Ortiz. Aparatos Eléctricos. S.A. de C.V., Monterrey, N.L. México) at 40°C, and the leaves were separated and ground using an electric mill (Molino Del Rey, S.A. de C.V. San Nicolás de los Garza, N.L. México).

### 2.3. Preparation of Extracts and Fractions

All extracts were prepared from a batch of dry, ground *F. cernua* leaves (180 g) and divided into 12 15 g portions. From each portion, one or three crude extracts was obtained, as described below. The extracts were designated by capital letters: (**A**) is the decoction (DC) and (**B** to **E**) correspond to the ethanol extracts that were prepared by still maceration (StMC (**B**)), Soxhlet extraction (SXH (**C**)), ultrasound-assisted maceration (UsMC (**D**)), and shake-assisted maceration (SkMC (**E**)), respectively. 

Three extracts were obtained with StMC from a unique 15 g portion of leaf powder that was subjected to successive extractions with *n*-Hex (**F**), EtOAc (**G**), and EtOH (**H**). The same procedure was also followed to obtain **I**–**K** using SXH, **L**–**N** using UsMC, and **O**–**Q** using SkMC with *n*-Hex, EtOAc, and EtOH as successive extraction vehicles.

The fractions were obtained by liquid-liquid partition (LLP), DC (**A**), and EtOH extraction (**B** to **E**). Each crude extract was partitioned successively with *n*-Hex, EtOAc, and *n*-BuOH. These fractions were named using the originating extract plus a subindex in sequence—for example, *n*-Hex, EtOAc, and *n*-BuOH fractions from DC (**A**) were termed **A_1_**, **A_2_**, and **A_3_**, respectively.

### 2.4. Decoction

A batch of *F. cernua* leaves was prepared by DC in the traditional mode of preparation [11]. Fifteen grams of ground leaves was combined with 150 mL double-distilled water and boiled for 20 min. The DC was filtered twice—first through a cotton plug and then through filter paper (Whatman no. 1)—and the resulting aqueous extract (**A**) was freeze-dried and stored until use.

### 2.5. Generation of Crude Ethanol Extracts

Crude ethanol extracts were obtained by StMC, SXH, UsMC, or SkMC. StMC was performed by extracting the plant material three times, each time with 300 mL for 72 h of incubation (3 × 300 mL × 72 h. SkMC) and SXH were performed on a rotary shaker (PC Corning 6200, NY, USA) and a Soxhlet apparatus, respectively, and the plant material was extracted 3 × 300 mL × 24 h at 100 rpm. UsMC was performed in an ultrasonic bath (Cole Parmer 8853, Vernon Hill, IL, USA), and the plant material was extracted 3 × 300 mL × 24 h. Crude ethanol extracts were filtered twice—first through a cotton plug and then through filter paper (Whatman no. 1)—and the solvent was evaporated under reduced pressure.

### 2.6. Generation of Extracts with Solvents of Ascending Polarities

Individual 15 g portions dry leaf material were successively extracted with *n*-Hex, EtOAc, and EtOH by StMC, SkMC, UsMC, or SXH. These extracts were termed **F**–**Q**, as described above.

### 2.7. Fractionation of Decoction and Crude Ethanol Extracts by Liquid-Liquid Partition

Each crude ethanol extract (1.0 g) was suspended in 500 mL H_2_O : MeOH (3 : 2, v/v). The suspension was successively partitioned between *n*-Hex (3x, 2 : 1, 1 : 1, 1 : 1, v/v), EtOAc (3x, 2 : 1, 1 : 1, 1 : 1, v/v), and *n*-BuOH (1x, 1 : 4, v/v). The hexane and butanol fractions were simply separated, and the solvent was evaporated; the ethyl acetate fraction was washed with saturated NaCl solution, dried over anhydrous Na_2_SO_4_, filtered through filter paper, and concentrated under reduced pressure. 

#### 2.7.1. *Mycobacterium tuberculosis* Strains

Two strains were used in this study: *Mycobacterium tuberculosis* H_37_Rv (ATCC 27294 (SMtb)), sensitive to all five first-line antituberculosis medications (streptomycin, isoniazid, rifampin, ethambutol, and pyrazinamide), and *M. tuberculosis* CIBIN/UMF15:99 (RMtb), a clinical isolate that is resistant to these medications.

#### 2.7.2. Mycobactericidal Activity

The mycobactericidal activity of the extracts and fractions was measured as the minimal mycobactericidal concentration (MBC)—the minimal concentration of each extract or fraction that killed the entire culture in a 200 *μ*L microplate well—by microplate Alamar Blue assay (MABA), modified by Molina-Salinas et al. [1]. Briefly, reference SMtb or RMtb cultures were added to a sufficient volume of sterile Middlebrook 7H9 broth, supplemented to achieve a turbidity that was equivalent to that of McFarland's no. 1 standard. This suspension was further diluted (1 : 50) with the same culture medium immediately before use. The organic extracts, decoction, and their fractions were assayed in duplicate.

All tests were performed in sterile flat-bottomed 96-well microplates (each well held 200 *μ*L). Working *F. cernua* preparations (100 *μ*L) were assayed in a two-fold dilution series in Middlebrook 7H9 broth, ranging from 200 *μ*g/mL to 6.25 *μ*g/mL. Each microplate was incubated for 5 d at 37°C and 5% CO_2_ in a sealed plastic CO_2_-permeable bag (Ziploc; Johnson and Son, Racine, WI, USA). After 5 days of incubation, blue (metabolically inactive mycobacteria) or pink (metabolically active mycobacteria) color was developed by adding, to each microplate-well, 32 *μ*L of a solution made with 9 volumes Alamar Blue (Trek Diagnostic, Westlake, OH, USA and 1 volume Tween 80 (Sigma).

The minimal inhibitory concentration (MIC) corresponded to the highest dilution of each *F. cernua* preparation that inhibited the mycobacteria growth (corresponding to the blue well contiguous to the first pink well). The mycobacterial growth or its absence was certified by direct observation of each microplate well with an inverted microscope. This was performed by comparing the appearance of cultures in each experimental microplate well with the untreated controls, which were placed in the same microplate. The organic extracts, decoction, and their fractions that had a MIC ≤ 100 *μ*g/mL were considered to be active. Then, the active extracts were examined for their mycobactericidal activity.

Immediately after the MIC was determined, 5 *μ*L of the last well that contained a blue suspension was transferred to a new microplate that contained 195 *μ*L of fresh culture medium per well. During the transfer, special care was taken to maintain the original relative position of each inoculum in the new plate. The following cultures were also transferred to a new microplate: (a) 5 *μ*L of two untreated mycobacterial suspensions (they were reinoculated separately, in triplicate); (b) a two-fold dilution series of rifampin (2–0.06 *μ*g/mL) and ofloxacin (16–0.5 *μ*g/mL). Three wells were inoculated with 100 *μ*L of fresh inoculum, as in the MABA, and three additional wells were incubated with 200 *μ*L of culture medium only as negative controls. The microplates were incubated and developed with Alamar Blue, as in the MABA.

#### 2.7.3. Definitions

The minimal bactericidal concentration (MBC) corresponded to the minimal concentration (in *μ*g) of each *F. cernua* preparation that did not allow a shift in color in cultures that were reincubated in fresh medium—for example, the MBC is the concentration of an *F. cernua* leaf preparation, expressed in *μ*g/mL, that kills 6 × 10^5^ mycobacteria in each well (6 × 10^6^/mL). One mycobactericidal unit (MBU) is equivalent to the one MBC but is expressed in *μ*g of solid material.

Absolute mycobactericidal activity (AMA) is defined as the number of MBUs in the entire mass of solids in the whole extract or fraction in 15 g dry *F. cernua* leaves, calculated per the following sequence of equations.

#### 2.7.4. Calculations

**(A) To Determine the Solid Yields, in *μ*g, of the Decoction or Ethanol Extracts:**

(1) $$\text{TE}{\text{S}}_{\text{D},\text{EE}}=\frac{{\text{Y}}_{\text{D},\text{EE}}\times \left(15\times 10^{6}\right)}{100},$$

where TES_D,EE_ is the total extracted solids in decoction or ethanol extracts, expressed in *μ*g; Y_E_ corresponds to the yield of each extract, expressed as the percentage of the original weight of ground, dry *F. cernua* leaves that were used to start each procedure (15 × 10^6^ *μ*g (Table 1)).

**(B) To Determine the Solid Yields, in *μ*g, of Each Fraction Derived from the Decoction or Ethanol Extracts:**

(2) $${\text{TES}}_{\text{A}1\text{–E}3}=\frac{{\text{TES}}_{\text{D},\text{EE}}\times {\text{Y}}_{\text{A}1\text{–E}3}}{100},$$

where TES_A1–E3_ are the total extracted solids in each fraction and Y_A1–E3_ is the yield of fractions A1 to E3 with respect to the content of solids (*μ*g) in the originating fraction (Table 2).

**(C) To Estimate the Yield (in *μ*g) of Solids in Successive Fractions Obtained from a Unique Ground, Dry F. cernua Leaves:**

(3) $${\text{TES}}_{\text{F–Q}}=\frac{{\text{Y}}_{\text{F–Q}}\times \left(15\times 10^{6}\right)}{100},$$

where TES_F–Q_ corresponds to the total extracted solids in each fraction F to Q, and Y_F–Q_ is the yield of fractions F to Q, respectively (Table 3);

(4) $${\text{TES}}_{\text{F}}=\frac{{\text{TES}}_{\text{D},\text{EE}}\times {\text{Y}}_{\text{F}}}{100},$$

where TES_F_ is the total extracted solids in each fraction and Y_F_ is the yield of each fraction with respect to the content of solids in the originating fraction (Table 3);

(5) $${\text{TES}}_{\text{F–Q}}=\frac{{\text{Y}}_{\text{F–Q}}\times \left(15\times 10^{6}\right)}{100},$$

where TES_F–Q_ corresponds to the total extracted solids in each fraction F to Q, Y_F–Q_ is the yield of fractions F to Q, respectively (Table 3), and 15 × 10^6^ is the original weight of the ground, dry *F. cernua* leaves.

15 × 10^6^  is the original weight of the ground, dry *F. cernua* leaves in all of these equations;

(6) $$\begin{array}{c} \text{Total}\;\text{MBUs}=\text{AMA},\\ 1\;\text{MBU}=\text{MBC}, \end{array}$$

where MBC is the minimal bactericidal concentration, expressed in *μ*g;

(7) $$\begin{array}{c} \text{MBC}=1.0\;\text{MBU},\\ \text{AMA}=\frac{\text{TES}}{\text{MBC}}, \end{array}$$

where TES is the total solids in each preparation, expressed in *μ*g.

## 3. Results

### 3.1. Decoction and Crude Ethanol Extracts from *F. cernua* Leaves

Table 1 shows the results of the decoction and the four ethanol extraction methods. SXH (**C**) and SkMC (**E**) generated the highest yields of material; DC (**A**) and UsMC (**D**) were less efficacious, 1.3- and 1.4-fold lower than yields of E, respectively. 

Regarding anti-*M. tuberculosis* activity, decoction (**A**) was inactive against the SMtb and RMtb strains, against both of which the ethanol extracts (**B**–**E**) had poor mycobactericidal activity. RMtb was twice as susceptible to all ethanol fractions.

### 3.2. Yields and Mycobactericidal Activity of Fractions from Decoction and Crude Ethanol Extracts

The yields and mycobactericidal activity of fractions by liquid-liquid partition of the decoction (**A**) and ethanol (**B**–**E**) crude extracts (Table 1) are listed in Table 2. The mycobactericidal activity resided primarily in the low-polarity fractions (**A_1_**, **B_1_**, **C_1_**, **D_1_**, and **E1**). The *n*-hexane fractions from the ethanol extract with SXH (**C_1_**) and DC (**A_1_**) produced the highest mass of solids, followed by UsMC (**D_1_**), StMC (**B_1_**), and SkMC (**E_1_**). The difference in yield between (**C_1_**) or (**A_1_**) and (**E_1_**) was 7% (1.5-fold). In contrast, the mycobactericidal activity of all *n*-hexane fractions increased significantly compared with their respective extracts. 

The changes in mycobactericidal activity that were observed during the partition of DC were the most dramatic—the fraction that had the highest activity was obtained (**A_1_**) from an inactive crude extract (**A**). The mycobactericidal effect of **A_1_** against SMtb and RMtb was 2- and 4-fold higher, respectively, than that of *n*-hexane fractions from the crude ethanol extracts (**B_1_**, **C_1_**, **D_1_**, or **E_1_**). The mycobactericidal activity of *n*-hexane fractions from the crude ethanol extracts against SMtb increased 4- and 2-fold against SMtb and RMtb, respectively. 

In a subsequent extraction with EtOAc, the yields and mycobactericidal activity of the resulting fractions varied more widely than those obtained with *n*-Hex. The fractions that had the lowest and highest content of solids came from DC (**A_2_**) and StMC (**B_2_**), respectively (24.7% difference between A_2_ and B_2_). In contrast, lower mycobactericidal activity was observed in all ethyl acetate fractions (**A_2_**–**E_2_**) with respect to those of *n*-*hexane.* The ethyl acetate fractions from **B** and **D** crude extracts maintained their mycobactericidal potency against RMtb while that of fractions from **A**, **C**, and **E** fell 2- to 4-fold against SMtb. Notably, ethyl acetate fractions from crude extract **B** had 4-fold lower mycobactericidal potency against SMtb versus its corresponding *n*-hexane fraction, but its activity against RMtb was maintained. Again, RMtb was more sensitive than SMtb to several ethyl acetate fractions (**B_2_**, **D_2_**, and **E_2_**). The *n*-hexane fraction **E_1_** lost its mycobactericidal activity against SMtb but maintained some activity against RMtb (Table 2).

The last serial extraction, performed with *n*-BuOH (**A_3_**–**E_3_**), generated fractions that had the lowest content of solids, ranging this from 6.7% (**E_3_**) to 17.7% (**A_3_**) of their respective extract (Table 2).

In contrast to the ethyl acetate fractions, the mycobactericidal activity of *n*-butanol fractions was uniform and weak: 100 *μ*g/mL against SMtb or 50 *μ*g/ML to 100 *μ*g/mL against RMtb. 

Material that was extracted with *n*-BuOH (**B_3_** to **E_3_**, Table 2) had the same mycobactericidal activity as crude ethanol extracts against SMtb (Table 1, **B** to **E**); 50% RMtb sensitivity was observed in fractions **B_3_**, **C_3_**, and **E_3_** (Table 2); **D_3_** showed the same antimycobacterial activity as extract **D** (Table 1).

### 3.3. Yields and Mycobactericidal Activity of Successive Extractions of *F. cernua* Leaf Powder with Solvents of Ascending Polarity by Various Extraction Methods

Table 3 shows that direct extraction of leaf powder with *n*-Hex was less efficient than with EtOH (Table 1); 16.5%, 21.8%, 11.2%, and 18.4% less solid material was obtained with StMC (**F**), SXH extraction (**I**), UsMC (**L**), and SkMC (**O**), respectively. The most efficacious method with *n*-Hex was SkMC (**O**), which extracted 11.9% less material than decoction (**A**; Table 1). Although less material was extracted by *n*-Hex versus EtOH or hot water (DC), its mycobactericidal activity against SMtb or RMtb was 2-fold higher than with EtOH; the extract that was obtained with *n*-Hex and StMC was 4-times more active against RMtb compared with the EtOH extract using the same method (Table 1).

Direct extraction of leaf powder with *n*-Hex generated 15.7%, 24.7%, 10.2%, and 13.8% fewer solids than starting the extraction from the crude ethanol extract (Table 2) with StMC (**F**), SXH (**I**), UsMC (**L**), or SkMC (**O**), respectively. The most productive method that used *n*-Hex directly was SkMC (**O**), yielding 21% less material than decoction by *n*-Hex fractionation (**A_1_** (Table 2)). 

The next extraction of residual material from the direct extraction with *n*-Hex was performed with EtOAc (Table 3), yielding 3.9% (**M**) to 4.8% (**G**). The mycobactericidal activity of material obtained with EtOAc after direct extraction with *n*-Hex (G, J, M, and P, using StMC, UsMC, and SXH, resp., (Table 3)) varied widely. 

Tables 2 and 3 show the following findings: (1) mycobactericidal activity (estimated as MBC) of G was 2-fold higher than the mycobactericidal activity of B_2_ against both SMtb and RMtb, using StMc; (2) the mycobactericidal activity of J was equal to that of C2 against SMtb and two-fold higher against RMtb, using SXH; (3) the mycobactericidal activity of M was two-fold lower than that of D2 against both SMtb and RMtb, using UsMC, and (4) the mycobactericidal activity of P was not comparable with that of E2 against SMtb and two-fold higher than that of E2 against RMtb, using SkMC.

The third sequential extraction with EtOH (Table 3) was slightly more productive than fractionation with *n*-BuOH (Table 2), extracting with StMC extract H contained 11.11% more solids fraction **B_2_**. Using SXH, 1.5% more solid material was extracted in **K** than in **C_3_**, and, with SkMC, EtOH extracted 10.4% more solid material in **E** than *n*-BuOH in E_3_. On the other hand, UsMC extracted 5.8% fewer solids with EtOH in N (Table 3) than with *n*-BuOH in D_3_ (Table 2).

Mycobactericidal activity against SMtb did not change with respect to the material that was extracted with EtOAc, but activity against RMtb doubled with StMC, SXH extraction, and SkMC. In contrast, mycobactericidal activity fell 50% with UsMC. 

Table 4 shows that although DC was inactive against SMtb and RMtb, the sum of the AMA values of its three fractions (**A_1_**, **A_2_**, and **A_3_**) was higher than the AMA of any crude ethanol extract (**B**–**E**) or the ΣAMA of its correspondent fraction set. 

In general, the NAMAs (normalized absolute mycobactericidal activities) of fraction sets that were derived from ethanol extracts (**B_1_**–**B_3_** to **E_1_**–**E_3_** (0.25–0.59)) were higher than their corresponding extracts (**B**–**E** (0.32–0.44)). The most active fraction set was prepared with a Soxhlet device ((**C_1_**–**C_3_**) NAMA = 0.59).

We observed similar results to those of Table 4 using RMtb as the target (Table 5), with two major differences: (a) the range in NAMA (0.36–0.50) of the fraction sets (**A_1_**–**A_3_** to **E_1_**–**E_3_**) was considerably narrower than the corresponding data in Table 4; the NAMAs of three of them were similar (StMC (B_1_–B_3_), 0.50; SXH (**C_1_**–**C_3_**) and SkMC (**E_1_**–**E_3_**), 0.49); and (b) the NAMA (1.02) that corresponded to the set of preparations that were obtained by sequential extraction with StMC (**F**–**H**) was comparable with that of the DC fraction set (**A_1_**–**A_3_**). Notably, the NAMA of the same extracts (**F**–**H**) was 2.75-times more active against RMtb (Table 5) than against SMtb (Table 4).

Excluding the NAMA of **F**–**H** against RMtb, the range in NAMA of the preparations that were obtained by sequential extraction (**I**–**Q**) was 0.42–0.67. 

Regarding the extraction efficacy of the solvents, *n*-Hex yielded the best results, followed by EtOAC. The DC hexane fraction (**A_1_**) contained 92.62% of the anti-RMtb activity of the **A_1_**–**A_3_** set (Table 5); the anti-SMtb activity in this fraction was 37.45% lower (Table 4). In this case, EtOAC (fraction **A_2_**) was slightly less effective than *n*-Hex, generating preparations that had 41.18% and 55.15% of the anti-SMtb activity, respectively (Table 4). 

Table 5 shows a similar pattern for the anti-RMtb activity of fraction **D_2_** (obtained with the aid of ultrasound (the AMA of **D_1_** was 44.58% and that of **D_2_** to 45% of the ΣAMA of **D_1_**–**D_3_**). The anti-RMtb of fraction **B_2_** (obtained with EtOAc and StMC) was higher (57.84%) than that of B_1_ (obtained with *n*-Hex). The range in anti-SMtb and anti-RMtb activities of fractions from ethanol extracts that were obtained with *n*-Hex (**B_1_**, **C_1_**–**D_1_**–**E_1_**) was 61.29% to 93.13% (Table 4) and 35.91% to 81.47% (Table 5), respectively. 

Anti-SMtb and anti-RMtb activities in fractions **B_1_**, **C_2_**, **D_2_**, and **E_2_** (obtained with EtOAc) ranged from ND (not detected) (**E_2_**)) to 61.29% and 15.52% to 62.10%, respectively.

For the preparations that were obtained by successive extractions with solvents of ascending polarity, *n*-Hex was the most efficient; anti-SMtb and anti-RMtb activities ranged from 40.72% to 70.99% and 48.34% to 78.58%, respectively (data calculated with respect to the ΣAMA of the **F**–**H**, **I**–**K**, **L**–**N**, and **O**–**Q** sets; Tables 4 and 5). 

The least efficient solvent for extraction of anti-*M. tuberculosis* material from DC or ethanol crude extracts was ethanol. In the DC preparation, anti-SMtb and anti-RMtb activities were 3.65% and 3.05%, respectively. The anti-SMtb and anti-RMtb activities of the ethanol fractions ranged from 3.74% to 6.89% and 2.0% to 9.96%, respectively.

With regard to successive extractions using solvents of ascending polarity, in general, EtOH was more efficacious than EtOAc in extracting material that had anti-SMtb and RMtb activities directly from ground, dry leaves. The AMA ranges of the material that was extracted with EtOAc and with EtOH against SMtb (expressed as a percentage of the ΣAMA of each set (**F**–**H**, **I**–**K**, **L**–**N** and **O**–**Q**)), were 11.55% to 21.72 and 16.98% to 37.56%, respectively. Against RMtb, the ranges were 9.92% to 26.74% (EtOH) and 11.55 to 35.93% (EtOAc; data calculated with respect to ΣAMA of each set of extracts).

## 4. Discussion

We have shown that decoction of *F. cernua* leaves is more effective than four of the most frequently used pharmacognostic methods in extracting metabolites that possess mycobactericidal activity against SMtb and RMtb *M. tuberculosis* strains—except for sequential extraction by StMC using solvents with ascending polarity whose efficacy equaled that of DC. 

As far as we know, the ability of DC and subsequent fractionation with solvents of ascending polarity to extract anti-SMtb and anti-RMtb has never been used with any medicinal plant.

DC fraction set had the highest NAMA against RMtb—equivalent to the NAMA of the most active anti-SMtb set—despite fact that DC did not have any activity against *M. tuberculosis,* as has been reported for *F. cernua* and other medicinal plants [1, 2, 4]. Furthermore, the sole *n*-Hex fraction contained more than 92% and 55% of the anti-RMtb and SMtb activities, respectively, of the DC fraction set. The AMA of DC *n*-Hex was higher than that of the *n*-Hex fractions from any of the ethanol extracts. These findings suggest that (1) DC, followed by fractionation with *n*-Hex, is more efficacious than ethanol extraction alone or followed by *n*-Hex fractionation, regardless of pharmacognostic method; (2) SMtb is more resistant than RMtb to *F. cernua* metabolites in the DC *n*-Hex fraction.

DC fractionation produced material having 41% more anti-SMtb activity than the most efficient method (extraction with ethanol, assisted with a Soxhlet device) and nearly 50% of the anti-RMtb activity in the fraction sets that were derived from the more bioactive ethanol extracts, generated by StMC, SXH, or SkMC. Moreover, the sole *n*-Hex DC fraction harbored 46% more anti-RMtb activity than any fraction set derived from ethanol extracts. 

Regarding the sequential extraction with solvents of ascending polarity, DC fractionation produced material having 60% more anti-SMtb activity than the most productive method (SkMC). In the case of the anti-RMtb, DC fractionation yielded a pattern similar to that observed against SMtb, with a unique exception: the NAMA of SkMC fraction set against RMtb was practically equal to the NAMA of DC fraction set.

It is intriguing that *F. cernua* metabolites are inactive when dissolved in boiling water, and, after being submitted DC to fractionation (primarily with *n*-hexane), these molecules exhibit a very strong antituberculosis effect in MABA solutions, which are rich in ions. 

We propose that anti-SMtb and anti-RMtb metabolites in *F. cernua* leaves are amphipathic (possibly polar lipids), because they were extracted well with hot water and *n*-Hex, which represent both extremes of polarity. Water and *n*-hexane have dielectric constants of 80 and 2, respectively [12]. Generally, polar solvents have a high dielectric constant and *vice versa*. 

Assuming that the *F. cernua* antituberculosis molecules are amphipathic, the compounds of interest might progress from an inactive to an active form. This happens after being submitted successively to boiling water, *n*-Hex, and an aqueous solution, rich in ions (MABA's). It is well known that the molecular and supramolecular structures of amphipathic compounds are strongly influenced by water and aqueous solutions rich in ions [13]. Thus, when the putative antituberculosis amphipathic molecules are passed into a hydrophobic medium (*n*-Hex), these possibly change to an intermediate conformation (not tested); when they are returned to a hydrophilic environment, these are modified again, producing active molecules or active suprastructures.

As Mexican people use a decoction of *F. cernua* leaves as an antituberculosis remedy [11], the abundant but inactive metabolites in this preparation must be activated, not by *n*-Hex, but by a metabolic pathway, as occurs with isoniazid [14], or during their absorption or transport. 

Notably, RMtb was 2-times more susceptible than SMtb to the *n*-hexane fraction of decoction. In contrast, RMtb was 8-times more resistant than SMtb to the DC-EtOH fraction. Nevertheless, this phenomenon did not occur with fractions that were derived from the ethanol extracts or with preparations from the successive extractions with solvents having ascending polarity. These data suggest that hot water, but not ethanol, can extract two classes of metabolites from *F. cernua* leaves: agents who are more hydrophobic and active against RMtb and compounds that are less hydrophobic and more active against SMtb. These putative classes of metabolites must be isolated and characterized. 

In conclusion, decoction of *F. cernua* leaves combined with *n*-Hex fractionation is more efficient than currently used pharmacognostic methods in extracting active metabolites against *M. tuberculosis.* DC can extract a compound that has valuable activity against RMtb with higher success than any other procedure. Our findings support the use of DC of *F. cernua* in Mexican traditional medicine to treat respiratory infections and engender the opportunity to treat patients who have been infected by multidrug-resistant *M. tuberculosis* strains.

## Figures and Tables

**Table 1:** Yield and mycobactericidal activity of crude extracts of *Flourensia cernua* leaf obtained with different extraction methods.

Extraction method	Solvent	Yield^a^	MBC^b^ (*μ*g/mL)		Resulting extract
			SMtb^c^	RMtb^d^	
DC	W	24.4	>100	>100	A
StMC	EtOH	25.5	100	50	B
SXH	EtOH	31.3	100	50	C
UsMC	EtOH	22.7	100	50	D
SkMC	EtOH	30.9	100	50	E

^a^Recovering percentage with respect to 15 g dry ground leaves (%w/w);  ^b^mycobactericidal activity MBC was expressed as the minimal bactericidal activity (MBC) that killed 100% of mycobacteria. ^c^SMtb *M. tuberculosis* H37Rv strain sensitive to all five fist-line antituberculosis drugs; ^d^RMtbCIBIN/UMF15:99, resistant to the above drugs. DC: decoction, StMC: still maceration, SXH: Soxhlet extraction, UsMC: ultrasound-assisted maceration, and SkMC: shake-assisted maceration; W: water; EtOH: ethanol. Rifampin showed an MBC = 0.062 and 100 *μ*g/mL and ofloxacin MBC = 0.125 and 0.250 *μ*g/mL versus SMtb and RMtb, respectively.

**Table 2:** Yields and mycobactericidal activity of fractions obtained by liquid-liquid partition of decoction and ethanol crude extracts of *Flourensia cernua*.

Originating extract^a^	*n*-Hex			EtOAc			*n*-BuOH		
	Fraction (yield^b^)	MBC^c^ (*μ*g/mL)		Fraction (yield^b^)	MBC^c^ (*μ*g/mL)		Fraction (yield^b^)	MBC^c^ (*μ*g/mL)	
		SMtb^d^	RMtb^e^		SMtb^d^	RMtb^e^		SMtb^d^	RMtb^e^
A	A_1_ (33.5%)	12.5	6.25	A_2_ (12.5%)	6.25	50	A_3_ (17.7%)	100	100
B	B_1_ (24.7%)	25	25	B_2_ (42.7%)	100	25	B_3_ (5.5%)	100	100
C	C_1_ (34.2%)	25	25	C_2_ (37.9%)	50	50	C_3_ (10.6%)	100	100
D	D_1_ (25.3%)	25	25	D_2_ (25.8%)	50	25	D_3_ (11.3%)	100	50
E	E_1_ (22.7%)	25	12.5	E_2_ (34.6%)	>100	100	E_3_ (6.7%)	100	100

^a^Decoction (A) or crude ethanol extracts (B–E) obtained with different extraction methods (Table 1) were successively fractionated by liquid-liquid partition with *n*-hexane (*n*-Hex), ethyl acetate (EtOAc), and *n*-butanol (*n*-BuOH) and assayed for their mycobactericidal activity. ^b^Yield was expressed as the percentage of dry material recovered with respect to the corresponding originating extract A–E and then with respect to 15 g of ground dry leaves of *F. cernua*. ^c^Mycobactericidal activity was expressed as the minimal bactericidal activity (MBC) that killed 100% of mycobacteria. ^d^SMtb *M. tuberculosis* H37Rv strain sensitive to all five fist-line antituberculosis drugs; ^e^RMtb *M. tuberculosis* CIBIN/UMF15:99, resistant to the above drugs. In this experiment rifampin and ofloxacin were included as internal standards, and these showed the same MBC reported in the experiments from Table 1.

**Table 3:** Yields and mycobactericidal activity of *F. cernua* ground dry leaves extracts obtained by successive extraction with solvents of increasing polarity using different extraction methods.

Extraction method	*n*-Hex			EtOAc			EtOH		
	Extract (yield^a^)	MBC^b^ (*μ*g/mL)		Extract (yield^a^)	MBC^b^ (*μ*g/mL)		Extract (yield^a^)	MBC^b^ (*μ*g/mL)	
		SMtb^c^	RMtb^d^		SMtb^c^	RMtb^d^		SMtb^c^	RMtb^d^
StMC	F (9.0%)	50	12.5	G (4.8%)	50	12.5	H (16.6%)	100	50
SXH	I (9.5%)	50	25	J (4.1%)	50	25	K (12.1%)	100	50
UsMC	L (11.5%)	50	25	M (3.9%)	100	50	N (5.5%)	100	100
SkMC	O (12.5%)	50	25	P (5.5%)	100	50	Q (17.1%)	100	50

*n*-Hex: *n*-hexane, EtOAc: ethyl acetate, EtOH: ethanol. StMC: still maceration, SXH: Soxhlet extraction, UsMC: ultrasound-assisted maceration, SkMC: shake-assisted maceration. ^a^Expressed as the percentage of 15 g dry ground of *F. cernua* leaves. ^b^Mycobactericidal activity was expressed as the minimal bactericidal activity (MBC) that killed 100% of mycobacteria. ^c^SMtb *M. tuberculosis* H37Rv strain sensitive to all five fist-line antituberculosis drugs; ^d^RMtbCIBIN/UMF15:99, resistant to the above drugs. In this experiment, rifampin and ofloxacin were included as internal standards. These showed the same MBC than those determined in the experiments from Table 1.

**Table 4:** Absolute mycobactericidal activity against SMtb in *F. cernua* leaf preparations^a^.

Extract (solvent)	Fraction (solvent)	Extraction or fractionation method	AMA^b^ (MBU)	ΣAMA^c^	NAMA^d^
A (W)		DC	ND	NQ	NQ
	A_1_ (*n*-Hex)	LLP	98,088		
	A_2_ (EtOAc)	LLP	73,200		
	A_3_ (*n*-BuOH)	LLP	6,478	**177,766**	**1.0**

B (EtOH)		StMC	**38,250**		**0.22**
	B_1_ (*n*-Hex)	LLP	37,791		
	B_2_ (EtOAc)	LLP	16,333		
	B_3_ (*n*-BuOH)	LLP	2,104	**56,228**	**0.32**

C (EtOH)		SXH	**46,950**		**0.26**
	C_1_ (*n*-Hex)	LLP	64,228		
	C_2_ (EtOAc)	LLP	35,588		
	C_3_ (*n*-BuOH)	LLP	4,977	**104,793**	**0.59**

D (EtOH)		UsMC	**34,050**		0.19
	D_1_ (*n*-Hex)	LLP	34,459		
	D_2_ (EtOAc)	LLP	17,570		
	D_3_ (*n*-BuOH)	LLP	3,848	**55,877**	**0.31**

E (EtOH)		SkMC	**46,350**		**0.26**
	E_1_ (*n*-Hex)	LLP	42,086		
	E_2_ (EtOAc)	LLP	ND		
	(E_3_) (*n*-BuOH)	LLP	3,106	45,192	**0.25**

F (*n*-Hex)		StMC	27,000		
G (EtOAc)		StMC	14,400		
H (EtOH)		StMC	24,900	**66,300**	**0.37**

I (*n*-Hex)		SXH	28,500		
J (EtOAc)		SXH	12,300		
K (EtOH)		SXH	18,150	**58,950**	**0.33**

L (*n*-Hex)		UsMC	34,500		
M (EtOAc)		UsMC	5,850		
N (EtOH)		UsMC	8,250	**48,600**	**0.27**

O (*n*-Hex)		SkMC	37,500		
P (EtOAc)		SkMC	8,250		
Q(EtOH)		SkMC	25,650	**71,400**	**0.40**

^a^Obtaining of extracts and their fractions as well as their correspondent yields and MBC is described in the footnotes of Tables 1–3. ^b^AMA means absolute mycobactericidal activity and is expressed as the number of mycobactericidal units (MBU) contained in the total mass of solids from an extract or fraction. One MBU is equivalent to the minimal mycobactericidal concentration expressed in *μ*g. The total content of solids in all extracts was calculated from yields of each extract or fraction, considering that all of these were obtained from portions of 15 g leaf powder (Tables 1–3). The solid mass of fractions was calculated considering the total solids of their correspondent extract. All calculations were performed using the equations and definitions described in Section 2. ^c^ΣAMA is the sum of MUs in all fractions derived from decoction or ethanol extracts, or the sum of extracts that came from the same portion of leaf powder and obtained with the same extraction method but with a different solvent (*n*-Hex, EtOAc, and EtOH). ^d^NAMA means normalized absolute mycobactericidal activity. 1.00 = Sum of AMA in the decoction fractions A_1_–A_3_.   ND means not detected and NQ not quantifiable.

**Table 5:** Absolute mycobactericidal activity against RMtb in *F. cernua* leaf preparations^a^.

Extract (solvent)	Fraction (solvent)	Extraction or fractionation method	AMA^b^ (MBU)	ΣAMA^c^	NAMA^d^
A (W)		DC	ND	NQ	NQ
	A_1_ (*n*-Hex)	LLP	196,176		
	A_2_ (EtOAc)	LLP	9,150		
	A_3_ (*n*-BuOH)	LLP	6,478	**211,804**	**1.0**

B (EtOH)		StMC	**76,500**		**0.36**
	B_1_ (*n*-Hex)	LLP	37,791		
	B_2_ (EtOAc)	LLP	65,331		
	B_3_ (*n*-BuOH)	LLP	2,104	**105,226**	**0.50**

C (EtOH)		SXH	**93,900**		**0.44**
	C_1_ (*n*-Hex)	LLP	64,228		
	C_2_ (EtOAc)	LLP	35,588		
	C_3_ (*n*-BuOH)	LLP	4,977	**104,793**	**0.49**

D (EtOH)		UsMC	**68,100**		**0.32**
	D_1_ (*n*-Hex)	LLP	34,459		
	D_2_ (EtOAc)	LLP	35,140		
	D_3_ (*n*-BuOH)	LLP	7,695	**77,294**	**0.36**

E (EtOH)		SkMC	**92,700**		**0.44**
	E_1_ (*n*-Hex)	LLP	84,172		
	E_2_ (EtOAc)	LLP	16,037		
	E_3_ (*n*-BuOH)	LLP	3,106	**103,315**	**0.49**

F (*n*-Hex)		StMc	108,000		
G (EtOAc)		StMc	57,600		
H (EtOH)		StMc	49,800	**215,400**	**1.02**

I (*n*-Hex)		SXH	57,000		
J (EtOAc)		SXH	24,600		
K (EtOH)		SXH	36,300	**117,900**	**0.56**

L (*n*-Hex)		UsMC	69,000		
M (EtOAc)		UsMC	11,700		
N (EtOH)		UsMC	8,250	**88,950**	**0.42**

O (*n*-Hex)		SkMC	75,000		
P (EtOAc)		SkMC	16,500		
Q (EtOH)		SkMC	51,300	**142,800**	**0.67**

^a^Obtaining of extracts and their fractions as well as their correspondent yields and MBC is described in the footnotes of Tables 1–3. Definitions, acronyms, symbols, and calculations are depicted Section 2 or in the foot note of Table 4.
